# Myocardial infarction induced by caseous mitral annular calcification: a case report

**DOI:** 10.1093/ehjcr/ytad329

**Published:** 2023-07-24

**Authors:** Joseph Cosma, Julien Wain-Hobson, Cecilia Gobbi, Andrea Zuffi, Cédric Joret

**Affiliations:** Cardiovascular Institute of Caen, Saint Martin Private Hospital, 18 Rue des Roquemonts, 14000 Caen, France; Cardiovascular Institute of Caen, Saint Martin Private Hospital, 18 Rue des Roquemonts, 14000 Caen, France; Cardiovascular Institute of Caen, Saint Martin Private Hospital, 18 Rue des Roquemonts, 14000 Caen, France; Cardiovascular Institute of Caen, Saint Martin Private Hospital, 18 Rue des Roquemonts, 14000 Caen, France; Cardiovascular Institute of Caen, Saint Martin Private Hospital, 18 Rue des Roquemonts, 14000 Caen, France

**Keywords:** Heart failure, Myocardial infarction, Caseous mitral annular calcification, CMAC, Mitral stenosis, Case report

## Abstract

**Background:**

Caseous mitral annular calcification (CMAC) is a rare variant of mitral annular calcification. Symptoms can be related to mitral valvular dysfunction, arterial embolization, and transient aortic outflow tract obstruction. CMAC usually affects the posterior fibrous mitral annulus and is commonly diagnosed in elderly patients with a history of hypertension, dyslipidaemia, and renal failure.

**Case summary:**

A 68-year-old patient was transferred to our department for late presenting acute myocardial infarction and acute heart failure. Coronary angiography revealed a significant extrinsic compression of the circumflex artery. Transthoracic echocardiography revealed an ovoid calcified mass of 3.6 cm × 2 cm originating from the posterior mitral annulus causing moderate mitral stenosis as well as akinesia of the inferolateral wall, reduced left ventricle ejection fraction (35%), and a low-flow low-gradient severe aortic stenosis. Cardiac computed tomography scan confirmed the presence of a large calcified mass, inserted to the posterior mitral annulus evocating caseous necrosis. The patient underwent a double valve replacement with implantation of both aortic and mitral bioprostheses. Histopathology of the excised mass revealed a chronic mitral valve fibrocalcification with aseptic necrosis, consistent with a caseous calcification of the posterior mitral annulus.

**Discussion:**

Extrinsic coronary artery compression is a rare disease entity. We report a rather peculiar cause of extrinsic artery compression: CMAC inducing significant mitral stenosis and compressing the circumflex artery leading to myocardial infarction. To the best of our knowledge, this is the first case of extrinsic artery compression caused by CMAC.

**Learning points**

1. CMAC is a rare variant of degenerative mitral annular calcification, with a prevalence of 0.07% in the general population.
2. Mostly incidental, it may cause, however, mitral valvular dysfunction, embolization of caseous necrotic debris, and/or calcium and cholesterol particles, and transient aortic outflow tract obstruction.
3. CMAC can cause extrinsic coronary artery compression leading to myocardial infarction.

## Introduction

Caseous mitral annular calcification (CMAC) is a rare variant of degenerative mitral annular calcification (MAC), with a prevalence of 0.6% of all MACs and 0.07% in the general population.^1,2^ Generally, a benign condition, it may, however, cause mitral valvular dysfunction, embolization of caseous necrotic debris of calcium or cholesterol particles, and transient aortic outflow tract obstruction.^3^ CMAC usually affects the posterior fibrous mitral annulus.^4^ The exact pathogenesis is unclear: it is commonly diagnosed in elderly patients, especially in those with a history of hypertension, dyslipidaemia, hypercalcemia, and end-stage renal disease.^2^ This suggests that CMAC may be associated with atherosclerosis and altered calcium-phosphate metabolism.^1,5–7^

We report the case of a 68-year-old patient admitted to our department for late presenting acute myocardial infarction and acute heart failure with evidence of extrinsic circumflex coronary (Cx) compression by CMAC of posterior fibrous mitral annulus, due to its close anatomical proximity.

## Summary figure

**Figure:**
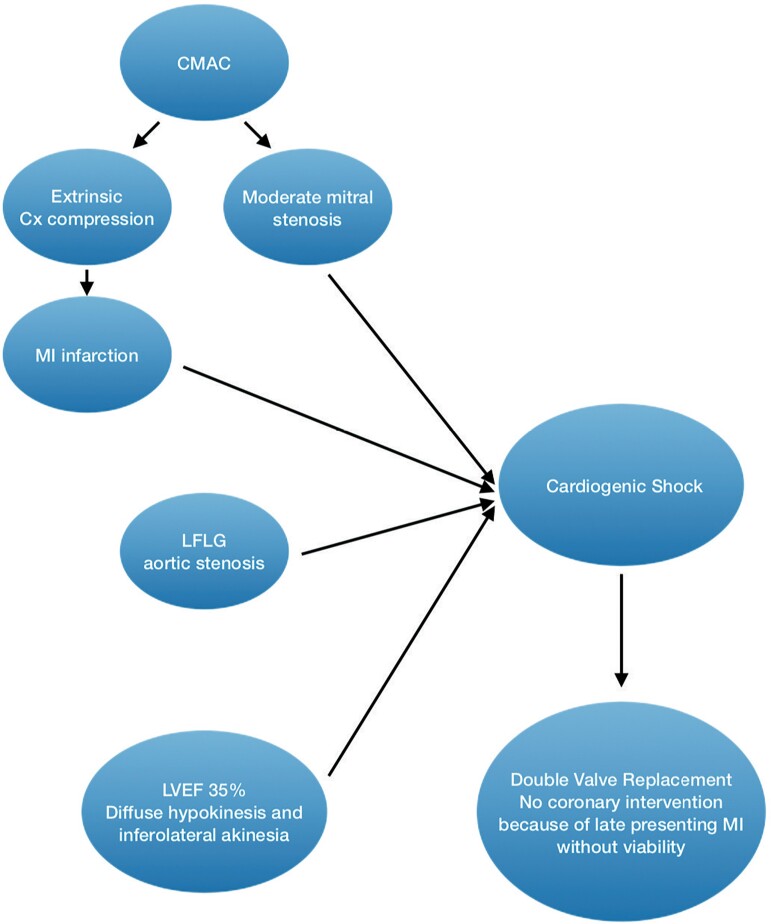


## Case presentation

**Figure 1:**
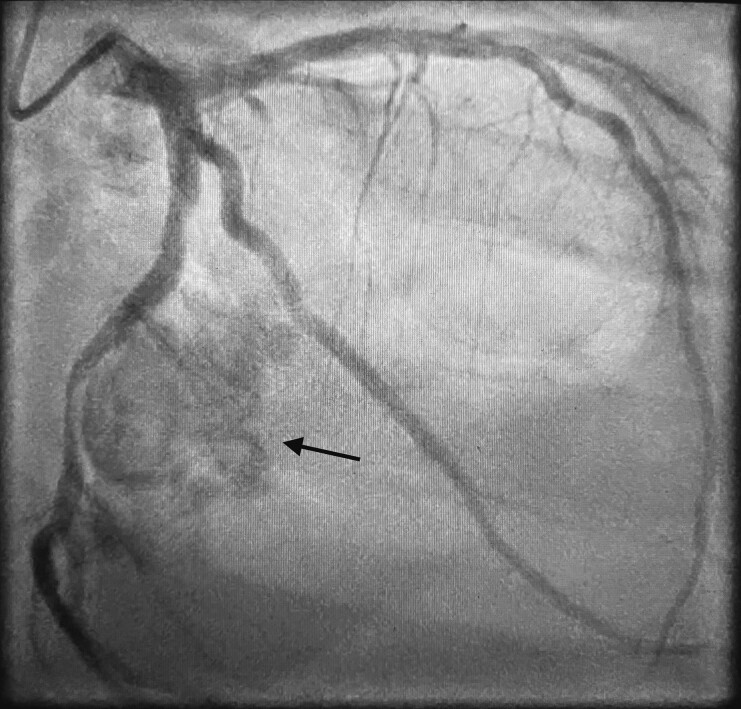
Coronarography showing extrinsic circumflex coronary artery compression (arrow).

**Figure 2:**
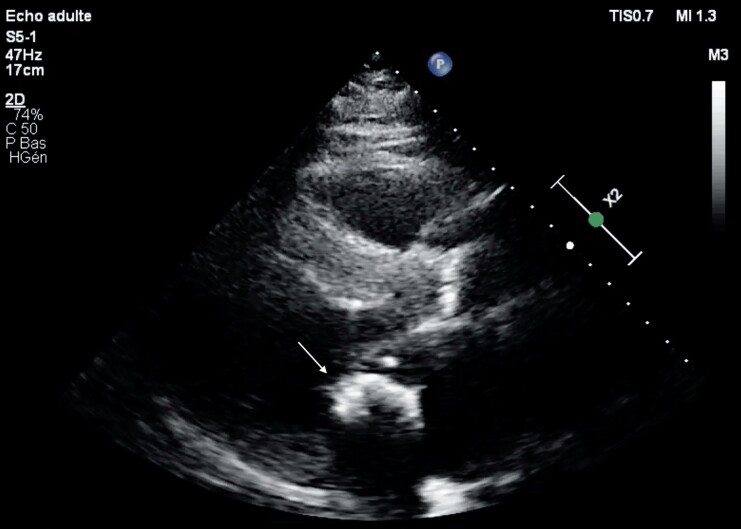
Transthoracic echocardiography parasternal long axis view showing a calcified mass originating from the posterior mitral annulus (arrow).

**Figure 3:**
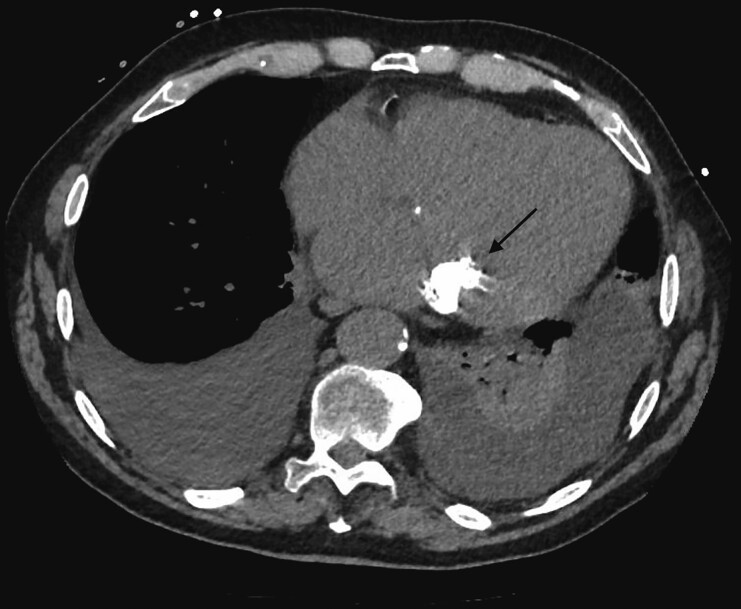
Thoracic computed tomography scan without contrast showing massive calcification of posterior mitral leaflet (arrow).

**Figure 4:**
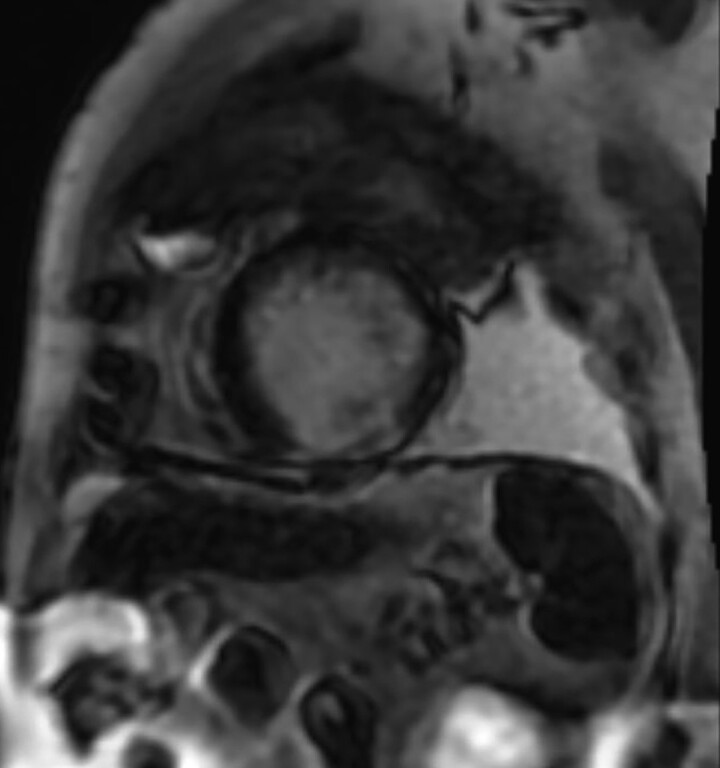
Cardiac magnetic resonance imaging. Short axis late-gadolinium enhancement imaging showing scar in the inferno-lateral wall.

**Figure 5:**
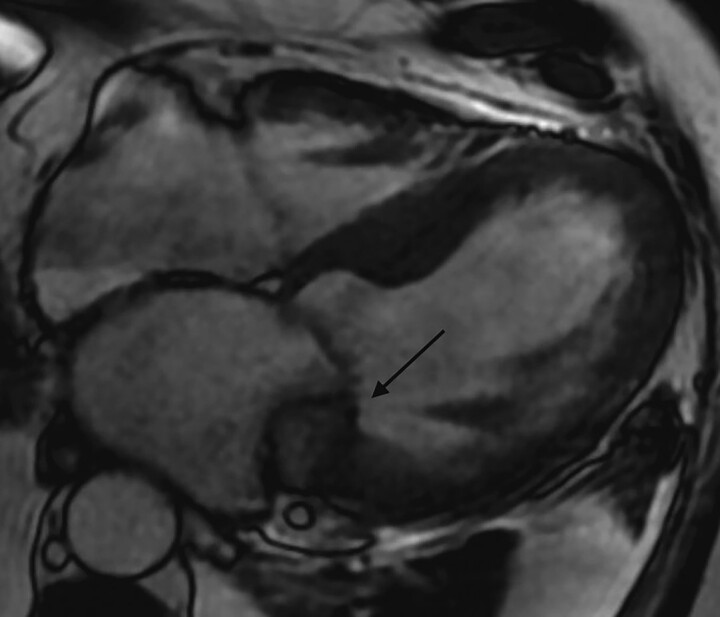
Cardiac magnetic resonance imaging. Late-gadolinium enhancement imaging showing hypointense mitral annulus mass with signal inhomogeneity (arrow).

**Figure 6:**
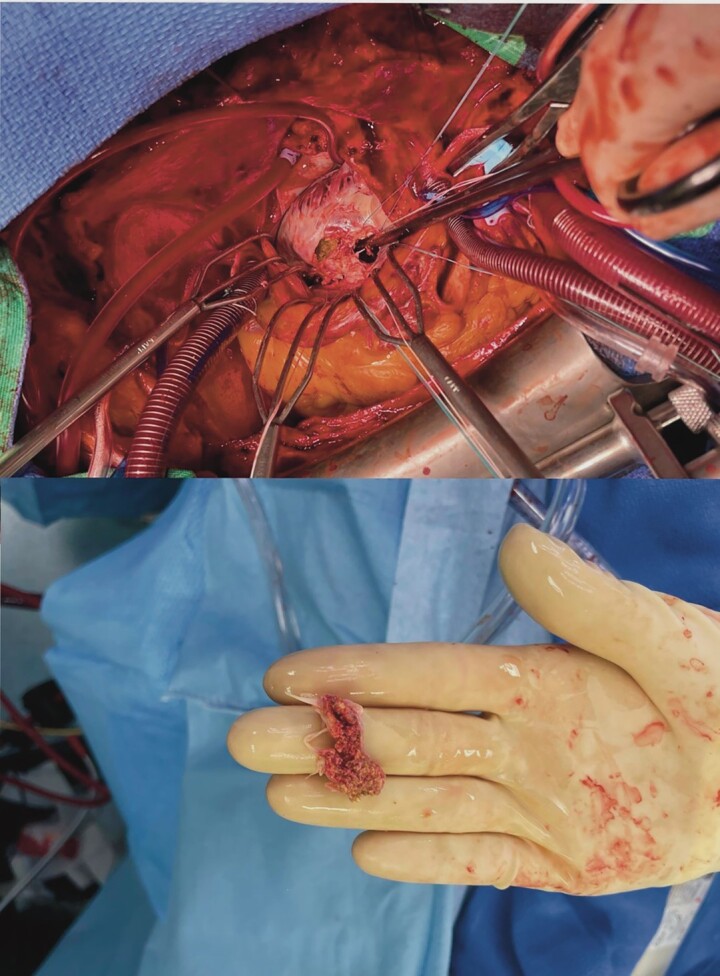
Extraction of the calcified mitral posterior leaflet.

A 68-year-old patient came to the emergency department for rapidly evolving dyspnoea without chest pain. He had no past cardiological medical history. On arrival, his heart rate was 130 b.p.m. and irregular, he was oxygen dependent, hypotensive (blood pressure 85/60 mmHg), and oligo-anuric. Physical examination revealed systolic and diastolic murmur, bilateral diffused crackles, and lower limb oedema. Electrocardiogram showed atrial fibrillation with a left bundle branch block. Blood sampling showed acute renal injury (creatinine 184 μmol/L, reference range 49–90 μmol/L), NT-ProBNP >35 000 pg/mL (reference range 0–125 pg/mL), troponin I 2685 pg/mL (reference range 0–16 pg/mL), and lactates 4.2 mmol/L (reference range 0.4–0.8 mmol/L). Diuretics and inotropic agent along with non-invasive ventilation were started with a gradual clinical and laboratory improvement. Coronary angiography revealed a single vessel disease with severe narrowing of Cx artery and an aspect of dynamic compression by an ovoid calcification (*Figure 1*, see supplementary files). Transthoracic echocardiography (TTE) revealed an akinesia of the inferolateral wall with reduced left ventricle ejection fraction (LVEF 35%) and significant calcification of the mitral and aortic valves conditioning a double valvular disease: a moderate mitral stenosis (mean Gradient 8 mmHg at heart rate 85 b.p.m., mitral valvular area 1.6 cm^2^) with a mild regurgitation and an ovoid calcified mass of 3.6 cm × 2 cm originating from the posterior mitral annulus (*Figure 2*, see supplementary files) together with a low-flow low-gradient severe aortic stenosis [stroke volume 31 mL/m^2^, mean gradient 35 mmHg, aortic valve area (AVA) 0.90 cm^2^, doppler velocity index (DVI) 0.22]. Cardiac computed tomography (CT) scan was performed and confirmed the presence of a large calcified mass, inserted into the posterior mitral annulus evocating caseous necrosis (*Figure 3*). The calcium score of the aortic valve at 5587 Agatston Units (AU) confirmed the severe aortic stenosis. Magnetic resonance imaging (MRI) revealed no viability of the basal and the mid segments of the inferolateral wall (*Figure 4*) and confirmed the presence of a low signal intensity mass in all sequences, reflecting the calcium content (*Figure 5*). The patient underwent a double valve replacement: sutureless Perceval XL (LivaNova) for the aortic valve and Epic 31 mm (Abbott) for the mitral valve plus a radiofrequency surgical ablation of the atrial fibrillation. The course of the hospitalization was marked by the gradual improvement of his clinical condition. The patient was discharged 15 days after cardiac surgery. The excised mass (*Figure 6*) was analysed and histopathology revealed a chronic mitral valve fibrocalcification with aseptic necrosis, consistent with a caseous calcification of the posterior mitral annulus. At 3 months, the patient was free of cardiovascular symptoms. TTE showed an improvement of the LVEF at 50% with inferolateral akinesia and good functioning of both bioprostheses.

## Discussion

The main causes of coronary extrinsic compression are the interarterial course^8^ or severe dilatation of the pulmonary artery causing left main compression. Other causes could be an abscess of the aortic root, aneurysm of the left aortic sinus, prosthetic pulmonary valve implantation, metastatic cardiac tumour, or ventricular pseudo aneurysm, among others.^9^ This is a report of a unique case of CMAC and inferolateral myocardial infarction probably caused by extrinsic compression of the Cx coronary artery. This latter vessel, after originating from the left main, goes past the left coronary sulcus heading towards the lateral and posterior walls of the left ventricle close by the posterior mitral annulus. Such anatomic conditions can explain how a calcified mass originating from the posterior mitral annulus can cause dynamic extrinsic compression. Increased narrowing of the Cx in systole observed on the coronary angiogram along with direct visualization of the compression of the artery by cardiac surgeons made our diagnosis more plausible in the absence of intravascular imaging. We did not perform IntraVascular UltraSound or Optical CT at first instance as the narrowing of the Cx was sub-occlusive, nor later, as MRI showed no residual viability in the inferolateral territory.

In this case, severe aortic stenosis led more easily to surgical treatment as there was neither severe mitral regurgitation nor criteria for severe mitral stenosis. It is important to remember that mitral valve replacement (MVR) in CMAC can be challenging and is associated with a high peri-operative mortality of up to 50% at one year in some series.^10^ The most common surgical technique is MVR associated with partial or complete surgical or ultrasonic debridement of calcified tissue. A multicenter global registry of elderly patients undergoing transcatheter MVR with the Edwards SAPIEN has also been published^11^ pointing to many possible therapeutic approaches.

## Conclusion

This is the first case where CMAC originating from the posterior mitral annulus led to extrinsic compression of the circumflex coronary artery and inferolateral myocardial infarction. Mitral valve replacement along with aortic valve replacement for associated aortic stenosis rapidly allowed improvement of the patient’s clinical condition.

## Supplementary Material

ytad329_Supplementary_Data

## Data Availability

The data underlying this article are available in the article and in its online Supplementary material.

## Lead author biography

Joseph Cosma was born in 1990 in Lecce. He got a degree in Medicine and Surgery in 2015 and completed his residency in Cardiology in 2020 at the University of Rome Tor Vergata. In 2021 he started a 2-year-fellowship in Interventional Cardiology at the Cardiovascular Institute of Caen. His main interests are interventional cardiology and echocardiography.

## Supplementary material

**Consent:** The authors confirm that written consent for the submission and publication of this case report, including images and associated text, has been obtained from the patient in line with COPE guidance.

**Funding:** No funding was received for this work.
