# Supplementary material for: Myocardial infarction induced by caseous mitral annular calcification: a case report
Source: Eur Heart J Case Rep. 2023 Jul 24;7(8):ytad329. doi: 10.1093/ehjcr/ytad329 (PMC10398422; doi:10.1093/ehjcr/ytad329)
Supplement: ytad329_Supplementary_Data [file ytad329_supplementary_data.zip › 23-00140R3_Approved additional author form.pdf]

Request for additional authors above article limits

This form should be used by the corresponding author if they wish to request that additional authors are required above the limits outlined by the journal. Please note that submission of this form does not guarantee agreement by the editors of EHJ-Case Reports to allow additional authors to be included.

All authors should be listed and their contributions defined. All authors are required to meet the 'authorship criteria' to be considered appropriate for inclusion. A reason for requesting more authors than than the authorship limit is required and should be selected from the dropdown list, please note the requirement to provide further details for these selections

**Article Title**	Myocardial Infarction induced by Caseous Mitral Annular Calcification: a case report		
**Article reference (if available)**	EHJ-CR-D-23-00140R3		
**Article Type**	Case report	**Author Limit**	4
**Corresponding Author name**	Joseph COSMA		
**Full author list (in the order you would like them to appear)**	Joseph COSMA, Julien WAIN-HOBSON, Cecilia GOBBI, Andrea ZUFFI, Cédric JORET		
**Reason for requesting additional authors**	Other (please describe below)		
**Additional details**	Dr Cecilia GOBBI was involved in patient care and actively partecipated in the writing of the case report.		

**Order**	**Author name**	**Author contribution**				**Meets authorship criteria**	**Notes**	**Author signature**
		**Involvement with patient care**	**Manuscript preparation**	**Final approval**	**Accountability**			
1	Joseph COSMA	Patient Care - Undertook	Drafting manuscript	Final approval of the manuscript	Agreement to be accountable for all aspects of the work	Yes		[illegible]
2	Julien WAIN-HOBSON	Patient Care - Undertook	Drafting manuscript	Final approval of the manuscript	Agreement to be accountable for all aspects of the work	Yes		[illegible]

3	Cecilia GOBBI	Patient Care - Undertook	Drafting manuscript	Final approval of the manuscript	Agreement to be accountable for all aspects of the work	Yes		Cecilia Gobbi
4	Andrea ZUFFI	Patient Care - Undertook	Critical revision of the manuscript	Final approval of the manuscript	Agreement to be accountable for all aspects of the work	Yes		[illegible]
5	Cédric JORET	Patient Care - Undertook	Critical revision of the manuscript	Final approval of the manuscript	Agreement to be accountable for all aspects of the work	Yes		[illegible]
